# The relationship between left ventricular mass index and body composition in new-diagnosed hypertensive patients

**DOI:** 10.1186/s40885-015-0033-6

**Published:** 2015-12-12

**Authors:** Sebnem Karakan, Bekir Inan

**Affiliations:** Department of Nephrology, Dr Abdurrahman Yurtaslan Oncology Training and Research Hospital, Ankara, Turkey; Department of Cardiovascular Surgery, Bezmialem University School of Medicine, Istanbul, Turkey

**Keywords:** Body composition, Left ventriculare mass index, Hypertension

## Abstract

**Background:**

Cardiovascular disease (CVD) is considered a public health burden and most common cause of mortality in all over the world. The latency time for developing CVD may be several decades. the objective of this study was to examine the relationship between body composition and Left Ventriculare Mass Index (LVMI) in newly diognosed hypertensive patients.

**Methods:**

We enrolled 120 new-diagnosed hypertensive patients (mean age 45 ± 8 years) who admitted to our nephrology clinic. Body fat percentage (BFP) was measured by bioelectrical impedance (BIA). Echocardiography examinations were performed for all patients.

**Results:**

Mean values of Waist hip ratio, Body mass ındex, Body fat percentage, Systolic blood pressure, Diastolic blood pressure were significantly higher for females than males (all p values <0.05). The female patients had higher LVMI than male patients (94.8 ± 13.1 vs 89.2 ± 14.6, *p* < 0.05). The study patients were divided into 3 groups according to their BFP defined by BIA. Group 3 patients, who exhibited higher body fat, had significantly higher BMI (*p* < 0,05), total leukocyte count (*p* < 0.05), CRP (*p* < 0.05), triglyceride (*p* < 0.05), and female predominance. Group 3 patients were statistically older than group 1 patients (46.2 vs. 40.6 years, *p* < 0.05). Additionally, LVMI levels were higher in Group 3 than Group 1 (*p* < 0.05) (Table 3). In logistic regression analysis, independent factors affecting LVMI were age, weight, gender and BFP (all p values were <0.05).

**Conclusions:**

BFP was associated with higher LVMI, in newly diognosed hypertensive patients. Its use results in significantly lower proportions of individuals with LVH in the population, in particular among hypertensive and the obese patients.

## Background

Cardiovascular disease (CVD) is considered a public health burden and most common cause of mortality in all over the world [1]. The risk increases continuously as left ventricular (LV) mass rises, with no indication of a threshold to clearly separate normal from abnormal LV mass dimensions [2]. When defining the range of normal LV mass, individual body size and composition must be taken into consideration because metabolic demand and perfusion needs vary with stature and determine the physiological adaptation of heart size. The latency time for developing CVD may be several decades [3]. Thus, early risk markers are needed to identify obese subjects at risk.

Obesity is a rapidly growing disease that is characterized by an excessive accumulation of adipose tissue [4]. LV mass was commonly indexed to body surface area (BSA) [5–8]. However, the assessment of Body Fat Percentage (BFP) in larger studies has long been hampered by technical impediments. The advent of bioelectrical impedance analysis (BIA) now provides a validated and easily applicable method of measuring BFP [9, 10]. BFP was analysed by BIA measurement.

Finally, the objective of this study was to examine the relationship between body composition and LVMI in newly diognosed hypertensive patients.

## Methods

We enrolled 120 new-diagnosed hypertensive patients (mean age 45 ± 8 years, 60 males) who were admitted to our nephrology clinic. Study exclusion criteria were antihypertensive (present or past) drug use, diabetes mellitus (current use of anti-diabetic medications, a fasting blood glucose level of more than 126 mg/dl, and/or a random blood glucose level of more than 200 mg/dl or hemoglobin a1c > 6.2 %), severe or secondary hypertension (≥210/130 mmHg), all endocrinological disorders (hyperparathyroıdısm, Addison's disease etc ..), renal failure (serum creatinine > 1.5 mg/dl), heart failure, peripheral or cerebral vascular disease, cancer and hepatic disease. After at least 5 min of rest in a sitting position, office BP was measured for all patients for bilateral arms using a sphygmomanometer with the appropriate cuff size. Three BPs were measured at least 5 min apart and the mean BP was used for analysis. Weight, height and waist circumference were determined for each subject; waist circumference was measured at the narrowest diameter between the costal margin and the iliac crest. Height (m) and weight (kg) were measured with the patient dressed in light clothing and without shoes. Body mass index (BMI) was calculated by the weight kilograms divided by the square of the height in meters. Blood samples were obtained after nocturnal fasting the same week as when 24-h ABPM was initiated. Blood chemistry were determined by standard methods. The study was approved by the local scientific ethics committee. The patients were enrolled after providing their informed consent.

All patients were analyzed by ambulatory blood pressure monitoring (ABPM) was recorded using the Oscar (Sun Tech Medical). The data were considered adequate when a minimum of 70 valid records were obtained in 24 h, with at least two records per hour during the nighttime. The following parameters were evaluated: average 24-h, daytime and nighttime systolic BP (SBP) and diastolic BP (DBP). Body fat percentage (BFP) was measured by bioelectrical impedance (BIA). Body composition and BIA data were obtained using the Metron BioScan 916 v3 analyzer. The first pair of electrodes was placed in the hand with the inner electrode attached to the dorsum of the wrist and the outer electrode attached to the dorsal surface of the third metacarpal bone. The second pair of electrodes was placed in the ipsilateral foot, with the electrodes placed on the anterior surface of the ankle and the dorsal surface of the third metatarsal bone. A single frequency, low-amplitude imperceptible current (0.7 mA at 50 kHz) was introduced via the outer electrodes. Body fat was calculated by subtracting FFM from total body weight (in kilograms).

### Echocardiography

Echocardiography examinations were performed with a 2D, M-Mode, pulse wave Doppler, and tissue Doppler echocardiography by using a Hewlett Packard, Sonos 7500, Andover (Massachusetts, USA) with a 2.8 MHz probe. Conventional echocardiography (M-mode and cPWD) measurements were performed according to American Society of Echocardiography guidelines [11]. Myocardial velocity profiles of the lateral mitral annulus were obtained by placing a 6 mm sample volume at the junction of the mitral annulus and lateral myocardial wall. Left ventricular mass (LVM) was calculated using the Devereux Formula [19]: LVM = 0.8(1.04(IVST + LVID + LPWT)^3^ – (LVID)^3^ + 0.6), where IVST = interventricular septal thickness, LVID = left ventricular internal dimension, and LPWT = left posterior wall thickness. The left ventricular mass index (LVMI) was calculated by the formula, LVM/(height)^2.7^ [11]. Statistical Analysis

Descriptive statistics are presented as mean ± SD. Continuous variables were tested to detect substantial deviations from normality by computing the Kolmogorov-Smirnov Z. The assumption of satisfactory normal distribution was met for all of the examined variables. Pearson correlation coefficients were used to explore the bivariate associations between examined variables and partial pearson correlation coefficients were used when appropriate. Stepwise multiple linear regression models were constructed using important covariates (weight, age, sex, BFP, waist) from correlation analyses to elucidate independent determinants of LVMI.

The minimum required sample size is calculated as 103 patients with anticipated effect size (f^2^) of 0.15, statistical power of 0.80 and 7 predictors. For all of the analyses, p levels < 0.05 were considered statistically significant. The data was analyzed using SPSS for Windows (version 16.0; SPSS Inc., Chicago, IL, USA).

## Results

A total of 120 new-diagnosed hypertensive patients (mean age 45 ± 8 years, 60 males) were included in the study. The demographic and clinical characteristics of the recipients are presented in Table 1. We investigated the relationship between body fat, inflammation, and laboratory parameters separately for males and females. Mean values of WHR, BMI, BFP, SBP, DBP were significantly higher for females than males (all p values <0.05). The female patients had higher LVMI than male patients (94,8 ± 13,1vs. 89,2 ± 14,6, *p* < 0.05).

**Table 1:** Demographic and laboratory features of study patients according to gender

Parameters	Female (*n* = 60)	Male (*n* = 60)	p value
*Demographic characteristics*			
Age (yr)	44 ± 9	43 ± 10	ns
BMI (kg/m^2^)	28.4 ± 1.8	26.3 ± 3.6	*p* < 0.05
WHR	88 ± 10	82 ± 9	*p* < 0.05
BFP %	38.8 ± 11,8	32,6 ± 12,0	*p* < 0.05
SBP(mm Hg)	142.8 ± 12.8	136.4 ± 10,8	*p* < 0.05
DBP (mm Hg)	88.5 ± 10.0	83,9 ± 11,1	*p* < 0.05
*Blood analysis*			
Glucose (mg/dl)	97.6 ± 9.5	99.5 ± 10.3	ns
Creatinin (mg/dl)	0.86 ± 0.14	0.86 ± 0.13	ns
LDL (mg/dl)	125.7 ± 32.3	118.5 ± 36.2	ns
Triglyceride (mg/dl)	164 ± 92	151 ± 111	ns
CRP (mg/dl)	3.1 ± 2.1	2.3 ± 1.7	ns
Hb (g/dl)	13.2 ± 0.9	14.3 ± 1.3	*p* < 0.05
Hct (%)	40.7 ± 3.8	42.2 ± 3.8	*p* < 0.05
Leukocyte count (per μL)	6.1 ± 1.9	5.9 ± 2.0	ns
PLT (per ml)	248.6 ± 60.0	272.8 ± 65.0	ns
LV mass/height^2.7^, g/m	94,8 ± 13,1	89,2 ± 14,6	*p* < 0.05
LV mass/BFP, g/kg	2,44 ± 0.99	2,73 ± 1.03	*p* < 0.05

*BMI* body mass ındex; *WHR* waist-to-hip ratio; *BFP* body fat percentage; *SBP* systolc blood pressure; *DBP* diastolic blood pressure; *LDL* low density lipoprotein; *CRP* C-reactive protein; *Hb* hemoglobulin; *Hct* hematocrite; *PLT* platelet

The study patients were divided into 3 groups according to their BFP defined by BIA (Table 2). Group 1 (BFP:<26 %, *n* = 30) comprised the patients in the lowest tertile of low BFP; group 2 (BFP = 26 to 48 %, *n* = 60), patients in the middle tertile; and group 3 (BFP > 48 %, *n* = 30), patients in the upper tertile. Group 3 patients, who exhibited ≥48 % of body fat, had significantly higher BMI (*p* < 0.05), total leukocyte count (*p* < 0.05), CRP (*p* < 0.05), triglyceride (*p* < 0.05) and female predominance. Group 3 patients were statistically older than group 1 patients (46,2 vs. 40,6 years, *p* < 0.05). Additionally, LVMI levels were higher in Group 3 than Group 1 (0.69 vs 0.65, *p* < 0.05) (Table 2).

**Table 2:** Comparison of clinical and laboratory values of patients according to BFP tertiles

Parameters	Group 1	Group 2	Group 3	*p*
	BFP ≤ 26 (*n* = 30)	BFP = 26-48 (*n* = 60)	BFP ≥ 48 (*n* = 30)	
Age	42.6 ± 4.2	44.4 ± 5.1	46.2 ± 3.1	*p* < 0.05
BMI	25.9 ± 2.1	27.1 ± 1.2	29.1 ± 1.6	*p* < 0.05
Triglyceride (mg/dL)	156 ± 101	157 ± 100	159 ± 112	*p* < 0.05
Leukocyte (per μL)	5.7 ± 2.1	5.9 ± 1.9	6.0 ± 2.0	*p* < 0.05
CRP	3.2 ± 1.7	4.2 ± 2.0	6.7 ± 2.1	*p* < 0.05
LVMI	88.9 ± 13.1	92.6 ± 10.1	95.2 ± 8.9	*p* < 0.05

*BMI* body mass ındex; *WHR* waist-to-hip ratio; *BFP* body fat percentage; *CRP* C-reactive protein; *LVMI* left ventruculare mass ındex

Mean LVMI were calculated as 91,4 ± 18,6 g/m^2^. Pearson correlation analysis was performed and BMI (r = 0.478, *p* = 0.01), systolic blood pressure (r = 0.621, *p* = 0.04), BFT (r = 0.493, *p* = 0.03) were positively and hemoglobin (r = -0.405, *p* = 0.02), ejection fraction levels (r = -0.288, *p* = 0.04) were negative correlated with LVMI. The criteria for the distinction of individuals with and without LVH differed considerably by sex. Partition value based on indexation to BFP was higher for women than for men.

In logistic regression analysis, we have converted continuous LVMI variable into categorical variable as high (above 91 g/m^2^) and low (91 and below g/m^2^). The cut-off value was arbitrarily chosen from the mean value of LVMI in the study population. Independent factors affecting LVMI were age, weight, and BFP (all p values were <0.05, Table 3).

**Table 3:** Results of stepwise multiple regression analysis for high LVMI (LVMI > 91 g/m2)

	β	p
BFP	0.280	0.001
+Age	0.188	0.036
+Weight, Age	0.268	0.020
+Sex, Weight, Age	0.112	0.129
+Waist, Sex, Weight, Age	0.292	0.116

*LVMI* Left ventricular mass index; *BMI* body mass index

p values <0.05 are significant

## Discussion

In our study, we investigated the influence of body fat composition on CV risk parameters in a group of hypertensive patients. Body fat distribution is associated with increased long-term mortality and morbidity risk, as shown in several recent studies [12, 13]. BIA is an adequate and easily accessible clinical tool for monitoring nutritional status in patients. We observed a direct correlation between bioimpedance measures and the traditional anthropometric and laboratory markers of cardiovascular risks in new-diagnosed hypertension patients.

BFP and BMI were significantly higher for females than males in our study (Table 1). The most important and drastic gender differences in BFP are related to reproductive functions that change through the age [14, 15].

We found that higher BMI and BFP were associated with higher LVMI. These data are in line with epidemiologic studies showing that Central body fat distribution is an independent risk factor for CVR factors. High BMI likely reflects high BFP rather than high muscle mass in our female, whereas men exhibited both increase in muscle mass and fat percentages in parallel to increase BMI. Skeletal muscle is the main site for insulin mediated glucose disposal, some investigators hypothesized that low muscle mass may be associated with insulin resistance and cardiovascular risks [16].

The associations of BFP were demonstrated by the multivariate analysis. Relation between body fat distrubition and CVD is in line with our prior findings in HD patients [17]. Furthermore, we demonstrated that this association is present in a population of hypertensive, non-diabetic patients. Several studies have reported that WHR was associated with albuminuria and BP both irrespective of BMI [18, 19]. In the current study, however, we have not found a robust association between WHR and albuminuria, possibly because of the very low albuminuria excretion rates, which reflect the strict inclusion criteria of these new diagnosed hypertensive patients.

High BFP group were older, and had higher BMI, and poor inflammatory profile in terms of higher levels of CRP. BFP was another factor that contributed to inflammation and vascular complications. An increase in adipose tissue results in the infiltration of macrophages and enhanced inflammation [20]. Moreover, it is associated with increased proinflammatory cytokines (IL-6, TNF-a) that contribute to atherosclerosis and CVD in high-fat composition population [20, 21]. Thus, inflammation play a role in classically initiated vascular injury, while at the same time, fat mass may independently initiate or propagate inflammation and vascular injury [22]. In this study, BMI and BFP increased together and cardiovascular risks were higher in high-BMI and high-BFP groups. On the contrary, increase in muscle mass could be protective against inflammation.

We have used LVMI instead of LVM in our study. Recent guideline advocate the use of indexed LV mass instead of measurements provided by septal thickness only. In a large cohort study of unselected adult outpatients referred to the echocardiography laboratory, the measurements of indexed LV mass applying the ASE/EAE recommended cut-offs yielded remarkable discrepancy in the diagnosis of LVH severity and offered prognostic information beyond that provided by septal thickness only criteria [23].

Several mechanisms may play a role in the deleterious effects of central fat accumulation on atherosclerosis, such as oxidative stress and inflammation by up regulation of pro-inflammatory adipokines and cytokines [24]. Furthermore, central fat is associated with dyslipidemia and inflammation parameters most likely in mutual interaction [25]. The combination of higher WHR and BFP indicate a high post-glomerular efferent arteriolar tone that can affect renal sodium handling [26]. We propose that BFP-indexed partition values of LVH be derived in other populations and that the prognostic significance of these values, including that in subgroups such as the obese, be evaluated in prospective studies.

The main limitation of our study is the small sample size for calculation of predictors in multiple regression analysis.

## Conclusions

In conclusion, BFP was associated with higher LVMI, in hypertensive persons. Of note, this association was dependent of BMI. BFP seems physiologically appropriate for LV mass indexation. Its use results in significantly lower proportions of individuals with LVH in the population, in particular among hypertensive and the obese patients
